# Female Gut and Genital Tract Microbiota-Induced Crosstalk and Differential Effects of Short-Chain Fatty Acids on Immune Sequelae

**DOI:** 10.3389/fimmu.2020.02184

**Published:** 2020-09-10

**Authors:** Emmanuel Amabebe, Dilly O. C. Anumba

**Affiliations:** Department of Oncology and Metabolism, University of Sheffield, Sheffield, United Kingdom

**Keywords:** gut, vagina, microbiota, metabolite, SCFA, dysbiosis, inflammation

## Abstract

The gut and genital tract microbiota of females represent very complex biological ecosystems that are in continuous communication with each other. The crosstalk between these two ecosystems impacts host physiological, immunological and metabolic homeostasis and vice versa. The vaginal microbiota evolved through a continuous translocation of species from the gut to the vagina or through a mother-to-child transfer during delivery. Though the organisms retain their physio-biochemical characteristics while in the vagina, the immune responses elicited by their metabolic by-products appear to be at variance with those in the gut. This has critical implications for the gynecological, reproductive as well as overall wellbeing of the host and by extension her offspring. The homeostatic and immunomodulatory effects of the bacterial fermentation products (short chain fatty acids, SCFAs) in the gut are better understood compared to the genital tract. While gut SCFAs prevent a leakage of bacteria and bacterial products from the gut in to circulation (leaky gut) and consequent systemic inflammation (anti-inflammatory/protective role); they have been shown to exhibit dysbiotic and proinflammatory effects in the genital tract that can lead to unfavorable gynecological and reproductive outcomes. Therefore, this review was conceived to critically examine the correlation between the female gut and genital tract microbiota. Secondly, we explored the metabolic patterns of the respective microbiota niches; and thirdly, we described the diverse effects of products of bacterial fermentation on immunological responses in the vaginal and rectal ecosystems.

## Introduction

The gut and genital tract microbiota of females represent very complex biological ecosystems. The gut microbiota is composed of about 10^13^–10^14^ bacterial cells (1, 2). While the upper reproductive tract (uterine cavity) was largely considered sterile (3, 4), the lower reproductive tract (cervicovaginal region) harbors trillions of bacteria (2). The origin of microorganisms that colonize the cervicovaginal space has been traced to the rectum, which also serves as a reservoir (5–7). The same bacterial species have been identified in the rectum and vagina of 36% of a cohort of 132 pregnant women with 68% of the species sharing identical genotypes (7). About 44% of bacterial species occurred in both the vaginal and rectal microbiota of these pregnant women despite being identified from either the vagina or rectum (7). This correlation in the microbial composition of both niches includes similarities in the species presence as well as the density of cells per bacterial species (5).

The gut and cervicovaginal microbiota can also interact with other organs and influence the health and disease balance of the host. Both microbiota can alter the homeostasis of other organs and systems of the body. Both microbiota interact with the immune system and modulate host immune responses. Though the gut microbiota has been studied extensively and described as akin to a solid organ with diverse functions, the complex and dynamic nature of the cervicovaginal microbiota is beginning to be appreciated (2) especially in relation to female health and reproductive outcomes (conception and birth) (8, 9).

Additionally, like in the gut, fecal and vaginal microbiota transplantation can be useful in the management of female genital tract disorders associated with changes in microbiota community composition (dysbiosis) (2, 10). The mechanisms underpinning these therapeutic initiatives are still being investigated. However, the commensal microbes as well as their metabolic by-products have been suggested to exhibit some antimicrobial and immunomodulatory activities that re-establish eubiosis and homeostasis (2, 10, 11). The implication of the differential immunomodulatory functions of the microbiota and the metabolites they elaborate is crucial to maintenance of region-specific homeostasis and the overall wellbeing of the host and by extension the offspring. This requires a more robust assessment. Therefore, this review was conceived to critically examine the correlation between the female gut and genital tract microbiota. Secondly, we explore the metabolic patterns of the respective microbiota niches; and thirdly, we describe the diverse effects of products of bacterial fermentation on immunological responses in the vaginal and rectal ecosystems.

## Gut Microbiota Composition

The gut microbiota is a complex heterogeneous ecosystem consisting of obligate anaerobes which are 2–3 times more than facultative anaerobes and aerobes (2). The bacterial population weighs up to 1.5 kg with more than 1100 species (1, 12). *Firmicutes* and *Bacteroidetes* constitute >90% of the gut microbiota, while *Actinobacteria, Proteobacteria, Fusobacteria, and Verrucomicrobia* contribute to the population by a lesser extent (1, 11–13). *Spirochetes* and *Lentisphaerae* are also present in small quantities (14). Some genera of these phyla are outlined in Table 1. The human gut microbiota also hosts other microbes such as archaea, yeasts, fungi, viruses, and protozoa (1, 11, 12), though their composition is still unresolved (1).

**TABLE 1:** Taxonomy of gut microbiota composition.

Phylum	Genus
Firmicutes	*Clostridium clusters*
	*Eubacterium*
	*Lactobacillus*
	*Bacillus*
	*Faecalibacterium*
	*Roseburia*
	*Ruminococcus*
Bacteroidetes	*Bacteroides*
	*Prevotella*
Proteobacteria	*Escherichia*
	*Shigella*
	*Helicobacter*
Actinobacteria	*Bifidobacterium*
	*Atopobium*
	*Corynebacterium*
Verrucomicrobia	*Akkermansia*
Fusobacteria	*Fusobacterium*

**Firmicutes* and *Bacteroidetes* constitute >90% of the gut microbiota (1, 11–13).*

Due to its complexity and dynamism, establishing the composition of an ideal and healthy gut microbiota has been challenging (2). A eubiotic gut microbiota is predominated by *Firmicutes* and *Bacteroidetes*, but deficient in inflammation-promoting *Proteobacteria* (12). However, the microbiota continues to fluctuate throughout life driven by mode of delivery (C-section or vaginally) (11, 15), age (11, 14), anatomical, and dietary/nutritional status (e.g., anorexic, lean, and obese nutritional status), environmental (e.g., familial composition, ethnicity, life-style, climate, occupation, stress etc.), pathological (e.g., gastrointestinal and systemic infections) and pharmacological factors (e.g., use of pre-/probiotics, antibiotics, prokinetics, and laxatives) (1, 2, 11). There are both intra- and inter-individual variations of gut microbiota (11, 14). The variability in bacterial species composition and density also spreads across various anatomical regions of the gastrointestinal tract (GIT) (Table 2) (2, 16–18). However, with the possibility of each individual sharing ∼40% of his/her gut microbial genes with at least half the general population, the existence of a functional core (core microbiome) has been postulated (19).

**TABLE 2:** Variability of predominant bacteria across the gastrointestinal tract.

Mouth, pharynx and distal esophagus	Stomach deficient of *H. pylori*	Jejunum and ileum	Cecum	Recto-sigmoidal colon
10^8^–10^9^ bacteria/ml	10^2^–10^4^ bacteria/ml	10^8^–10^10^ bacteria/ml	10^11^–10^12^ bacteria/ml	
*Streptococcus*	*Streptococcus*	*Streptococcus*	*E. coli*	*Bacteroides*
*Prevotella*	*Gemella*	*Staphylococcus*	*Enterococcus*	*Clostridium coccoides*
*Actinomyces*	*Actinomyces*	*Enterococcus*	*Lactobacillus*	*Clostridium leptum*
*Haemophilus*	*Prevotella*	*E. coli*	*Roseburia*	*Bifidobacterium*
*Granulicatella*	*Lactobacillus*	*Lactobacillus*	*Faecalibacterium*	*Alistipes*
*Rothia*	*Propionibacterium*	*Klebsiella*	*Eubacterium*	*Parabacteroides*
*Gemella*	*Staphylococcus*	*Bacteroides*	*Anaerostipes*	
*Veillonella*		*Veillonella*	*Ruminococcus*	
*Bifidobacterium*		Clostridium cluster XIVa		
*Propionibacterium*				
*Lactobacillus*				
*Bacteroides*				

*The genera are not listed according to their respective relative abundance in each region (2, 16–18, 56).*

The gut microbiota is involved in several immune, metabolic, and nutrients absorption functions that are integral to the host survival (1, 14). Importantly, there is interaction between gut microbiota and host immune system that ensures tolerance of commensal bacteria and antigens ingested with food, while maintaining the ability to identify and attack potential pathogens and prevent invasion and infection (2, 20). The gut microbiota also contribute to innate and adaptive immunity. Commensal bacteria and their products regulate the development and function of innate and adaptive immune cells. They prevent colonization by opportunistic pathogens by inhibiting their growth, nutrient depletion, pH modification, production of bacteriocins, and maintenance of intestinal epithelial barrier (11). Commensal bacteria also inhibit growth and colonization by pathogens by decreasing the expression of genes linked to virulence factors such as locus of enterocyte effacement (LEE) (12, 21), and regulatory effects on cell signaling pathways (11), e.g., bile acid regulation of immune cells via GPBAR1 and NRIH4 leading to repression of NF-κB proinflammatory activity (22).

The gut microbiota also influences digestion and absorption of nutrients, being involved in the extraction, synthesis, and absorption of polysaccharides, amino acids, lipids, vitamins, bile acids, and short chain fatty acids (SCFAs) (11, 14, 23). The immune and metabolic functions of the gut microbiota are essential for maintaining homeostasis such that dysbiosis has been associated with both extra-intestinal disorders (such as obesity, metabolic syndrome, type 2 diabetes, allergy, and asthma); and intestinal disorders such as colorectal cancer, inflammatory bowel disease (IBD), irritable bowel syndrome, and celiac disease (11, 12, 14). In addition, some central nervous system-related disorders including Alzheimer’s disease, Parkinson’s disease, autism spectrum disorders and hepatic encephalopathy have been related to gut microbiota dysbiosis (11).

## Female Genital Tract Microbiota

The female genital tract comprise of the lower (cervicovaginal) region and the upper (uterine cavity, fallopian tubes, and ovaries) region with variable bacterial relative abundance and diversity (Table 3). The normal physiological vaginal microbiota was originally described as homogenous, consisting of only Gram-positive bacilli of the *Lactobacillus* genus that emerge from the gut (8). Through the use of metagenomics techniques, the microbial composition of the cervicovaginal space has been characterized (24). Though the vaginal microbiota comprise of a large community of commensal and potential pathogens, the predominant species observed in healthy women are *Lactobacillus* (i.e., *Lactobacillus crispatus, Lactobacillus gasseri, Lactobacillus iners*, and *Lactobacillus jensenii*) (25). Other commensal anaerobic species with great propensity to transit to pathogens especially when lactobacilli are depleted include *Gardnerella, Prevotella, Megasphaera, Atopobium, Streptococcus, Mobiluncus, Mycoplasma, Peptoniphilus* etc. (2, 8, 26). *Lactobacillus* spp. and the other anaerobes exist in an inverse relationship (2, 27). The physiologic vagina microbiota contains about 10^6^–10^8^ bacterial cells/ml (28). More recent studies report up to 10^10^–10^11^ bacterial cells, and 10^2^–10^4^ (four orders of magnitude) higher abundance, but lesser diversity compared to the uterine microbiota (29, 30).

**TABLE 3:** Variability of predominant bacteria genera across the female reproductive tract.

Lower genital tract		Upper genital tract		
	
Vagina	Cervical canal	Endometrium	Fallopian tube	Ovary
Lactobacillus*	Lactobacillus*	*Lactobacillus*	*Acinetobacter*	*Acinetobacter*
*Gardnerella*	*Acinetobacter*	*Pseudomonas*	*Comamonas*	*Sphingomonas*
*Atopobium*	*Prevotella*	*Actinobacteria*	*Pseudomonas*	*Methylobacterium*
*Prevotella*	*Corynebacterium*	*Vagococcus*	*Pseudomonadaceae*	*Lactococcus*
*Streptococcus*		*Sphingobium*	*Vagococcus*	*Corynebacterium*
*Corynebacterium*		*Comamonadaceae*	*Comamonadaceae*	*Blautia*
*Gemella*		*Arthrobacter*	*Arthrobacter*	*Escherichia*
*Dialister*		*Dysgonomonas*	*Dysgonomonas*	*Lactobacillus*
*Sneathia*		*Shewanella*	*Shewanella*	*Trabulsiella*
*Megasphaera*		*Pseudomonadaceae*	*Pseudomonadaceae*	
*Mobiluncus*		*Delftia*	*Delftia*	
*Ureaplasma*		*Tissierellaceae*	*Sphingobium*	
*Mycoplasma*		*Sphingomonas*	*Sphingomonas*	
*Peptoniphilus*		*Erysipelotrichaceae*	*Erysipelotrichaceae*	
*Aerococcus*		*Erysipelothrix*	*Erysipelothrix*	
*Parvimonas*		*Blautia*	*Lactobacillus*	
*Eggerthella*		*Corynebacterium*	*Facklamia*	
*Pseudomonas*		*Staphylococcus*	*Tissierellaceae*	
*Bacteroides*			*Staphylococcus*	
*Fusobacterium*			*Micrococcaceae*	
			*Oxalobacteraceae*	
			*Ochrobactrum*	
			*Achromobacter*	
			*Bacteroides*	
			*Enterococcus*	
			*Stenotrophomonas*	
			*Brevibacterium*	
			*Coprococcus*	
			*Corynebacterium*	
			*Anaerococcus*	
			*Propionibacterium*	
			*Prevotella*	
			*Burkholderia*	

*Genera constituting >97% of the microbiota indicating low diversity in the lower genital tract but higher abundance up to four times that of the uterus. In the upper genital tract, the genera are not listed in order of their respective relative abundance in a healthy state, as this is still a subject of debate (29, 30, 47, 51, 117–120).

**FIGURE 1:**
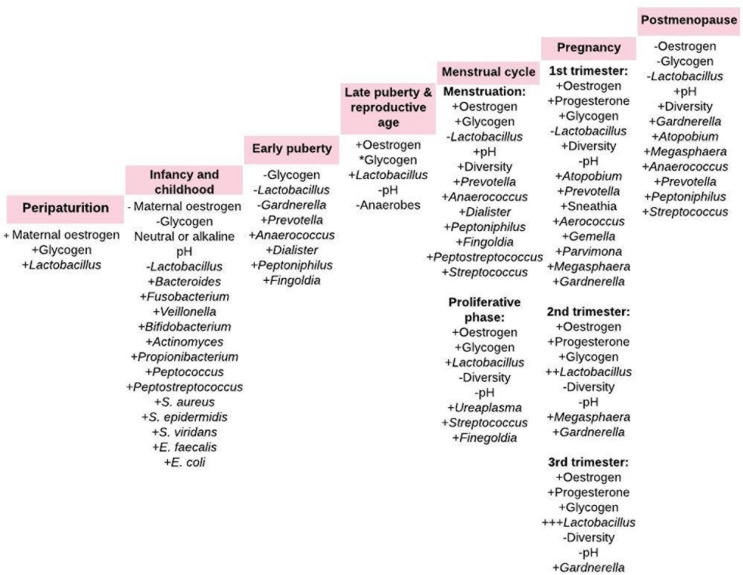
Vaginal microbiota composition from periparturition to postmenopause. The menstrual cycle comprise of uterine and ovarian cycle. The proliferative phase of the uterine cycle coincides with the follicular phase of the ovarian cycle, which overlaps menstruation. +, increase; –, decrease; *cyclical change in intracellular glycogen content (2, 26, 27, 31, 51).

The vaginal microbiome is a dynamic and closely regulated ecosystem (2, 27), which continuously evolves throughout the female lifecycle driven by hormonal changes (Figure 1) (31). Reversible changes also occur during menstruation and pregnancy. During menstruation, there is up to 100-fold decrease in *L. crispatus* and increase in *L. iners, Gardnerella vaginalis, Prevotella bivia*, and *Atopobium vaginae*. However, there is a more stable lactobacilliary vaginal microbiota during normal pregnancy. These changes are attributed to high estrogen and glycogen levels as seen in premenopausal women (especially during pregnancy) and decreased levels as seen during the menstruation (8). However, there are instances where these associations may be absent. For example, a recent pilot study in black adolescent women (14.3 ± 0.3 years) concluded that vaginal glycogen and not estradiol nor psychosocial stress is associated with vaginal microbiota composition (32). The vaginal microbiome is also affected by sexual intercourse, contraceptive devices, smoking, stress, douching and use of antibiotics, and probiotics (8, 9, 33–36).

The bacteria in the urogenital tract, most of which are uncultivable, constitute ∼1/10 of the total human microbiota. Despite the anatomical proximity of the cervicovaginal space and uterine cavity, the healthy uterine cavity has been believed to be “sterile” (3, 37) due to the “impermeable” barrier constituted by the cervical plug. This barrier prevents the ascent of bacteria from the vagina (38). This assertion has been challenged and it is currently argued that the cervical plug is not impermeable to bacterial flow from the vaginal tract; hence, the uterus may not be sterile (3, 39). Furthermore, bacteria may also reach the uterus through other channels including retrograde migration through the fallopian tubes and hematogenous transfer from the gut or oral microbiome through the placenta when the woman is pregnant (3, 40).

In support of the hematogenous microbial link is the association of periodontitis with increased risk of preeclampsia, intrauterine growth restriction, premature delivery, and delivery of low birth weight infants (41, 42). *Porphyromonas gingivalis* has been detected in both the amniotic fluid and the subgingival plaque of women in preterm labor (43). Similarly, the same strain of *Bergeyella* spp. which was absent in the vagina of a pregnant woman with clinical chorioamnionitis was identified in both her amniotic fluid and subgingival plaque (44). *Fusobacterium nucleatum*, an oral anaerobe, has also been identified in the placenta permitting colonization by other pathogens such as *E*scherichia *coli* (45). Changes in the gut/oral cavity microbiota (and not vaginal microbiota alone) may determine the risk of preterm birth (4, 46). However, it is noteworthy that the existence of a uterine and/or placental microbiota is still a subject of debate (47).

Similar to the relationship of gut microbiota and GIT cancers, the vaginal microbiome is also associated with female reproductive tract tumors. Especially in women with invasive cervical carcinoma, *Lactobacillus* spp. abundance and elevated vaginal pH negatively and positively correlates with high levels of cancer biomarkers in the cervicovaginal microecosystem, respectively. Dysbiotic vaginal microbiota deficient in lactobacilli with concomitant increase in pH is associated with persistent human papillomavirus (HPV) infection, cervical epithelial dysplasia and progression to invasive cervical carcinoma (48). Furthermore, vaginal microbiota dominated by *Prevotella, Streptococcus, Atopobium*, *Ureaplasma*, *Mobiluncus*; and deficient in lactobacilli was associated with increased predisposition to ovarian cancer or factors that influence its risk including age and *BRCA1* germline mutations (49).

The role of the vaginal microbiota in the protection/acquisition of sexually transmitted infections has been discussed previously (8).

## Gut-Vagina Microbiota Crosstalk

**FIGURE 2:**
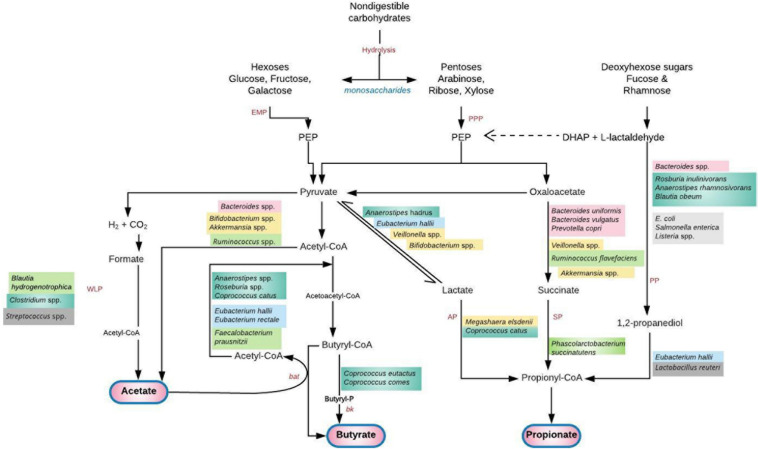
Saccharolytic fermentation of dietary non-digestible carbohydrates into short chain fatty acids (SCFAs) by gut microbiota. A cross feeding of intermediate metabolites between species exists. Acetate produced by *Bifidobacterium* spp. is utilized by *Firmicutes* (*F. prausnitzii*, *Roseburia*, *Anaerostipes*, *Eubacterium*) to produce butyrate (56). Lactate, succinate and 1, 2-propanediol do not usually accumulate to high levels in the colon of healthy adult humans due to their utilization by the propionate and butyrate-producing species (78, 82). Deficiency of these species and their metabolic by-products permits a “leaky gut” with transfer of bacteria and lipopolysaccharide to systemic circulation. A plausible hematogenous spread of these pathogen-associated molecular patterns to the genital tract or global induction of systemic inflammation can lead to genital tract infection/inflammation and unfavorable gynecological/reproductive outcomes (59). Furthermore, translocation of gut microbial species to the vagina and consequent high levels of vaginal SCFA production alters the vaginal microbiota leading to infection (94). *AP*, acrylate pathway; *bat*, butyryl-CoA:acetyl-CoA transferase; *bk*, butyrate kinase; *DHAP*, dihydroxyacetone phosphate; *EMP*, glycolytic Embden-Meyerhof-Parnas pathway; *PEP*, phosphoenolpyruvate; *PP*, propandiol pathway; *PPP*, pentose-phosphate pathway; *SP*, succinate pathway; *WLP*, Wood–Ljungdahl pathway. Adapted with permission from: Tungland (77).

Transfer of bacterial strains from the gut to vagina has been indicated (50). Both niches have been shown to harbor five common bacteria phyla – *Firmicutes*, *Bacteroidetes*, *Proteobacteria*, *Actinobacteria*, and *Fusobacteria* (20, 51). The Gram-positive bacilli *Lactobacillus* that dominate the vaginal microbiota in health is believed to originate from the gut (8). Lactobacilli are abundant in the gut (16), where they abet energy, metabolic and immunologic homeostasis (16, 20). The crosstalk of bacterial strains between the gut and vagina stimulates both local and systemic immune responses with attendant effect on the overall host physiology (Figures 1, 2) (2).

### Vertical Transmission of Microbiota

Vaginal delivery is associated with newborn gut microbiota that suggests a maternal vaginal source (3, 52). Infants born by vaginal delivery acquire gut microbiota similar to their mothers’ vaginal microbiota. Analysis of the meconium of newborns delivered vaginally reveal the presence of *Lactobacillus*, *Prevotella*, and *Sneathia* similar to the mother’s vaginal microbiota (52). The infant gut is also colonized by *Bifidobacterium longum*, *Bifidobacterium catenulatum*,(53) and other anaerobes such as *Faecalibacterium*, *Roseburia*, *Staphylococcus*, *Streptococcus*, *Atopobium*, *Akkermansia*, *E. coli*, *Bacteroides, Prevotella, Methanobrevibacter, Peptostreptococcus, Veillonella*, etc. (3, 15, 54, 55). Bifidobacteria, which are among the first colonizers of the human gut, comprise up to 90% of the total colon microbiota in vaginally delivered breast-fed infants in the first year of life (56). There is a strong correlation between the gut microbiota of newborns delivered vaginally and the microbial communities of the mother’s vagina (53). This is achieved through vertical transmission of microbiota from mother’s vagina to infant gut (54).

In contrast, the intestinal microbiota of C-section delivered neonates has lesser microbial richness and diversity (53, 57) with underrepresentation of *Escherichia*, *Shigella*, and *Bacteroides* species (57). However, the microbiota is dominated by *Staphylococcus*, *Corynebacterium*, *Propionibacterium* spp. acquired from the environment and mother’s skin microbiota (3, 52). Some C-section delivered infants have even shown non-maternal skin microbes (58, 59). Anal samples from infants delivered vaginally and by C-section but swabbed with their mother’s vaginal microbiota, were enriched with *Lactobacillus* and *Bacteroides*. On the other hand, C-section neonates not exposed to their mother’s vaginal microbiota lacked these microbes and instead harbored high amounts of *Clostridium* (60).

Because vertical mother-to-infant transmission of microbiota is important for host metabolism and immune development, infants born by C-section are at increased risk of chronic immune disorders including neonatal respiratory morbidity, bronchiolitis, respiratory syncytial virus infection, allergies (asthma, hay fever, and eczema), laryngitis, gastroenteritis, IBD, celiac disease, leukemia, neuroblastoma, atopic dermatitis, juvenile idiopathic arthritis, obesity, and type 1 diabetes (61, 62). This is somewhat due to decreased Th1 development and activity leading to decreased antibody and cytokine production, decreased phagocytosis and allergy-associated Th2 overactivity. Apart from not traversing the vagina to acquire its microbiota, there is absence of the labor-associated cytokine-mediated inflammatory response and inactivation of the hypothalamic-pituitary-adrenal axis resulting in reduced corticotropin-releasing hormone and cortisol, which are necessary for maturation of organs including the lungs, GIT, and immune system (50, 63).

### Gut-Vagina Microbiota and Immune Interaction

The microbiota (especially bacteria) and immune mediators in the gut and vagina are in close interaction. Oral administration of probiotics (viable microorganisms) (20) influence vaginal microbiota composition and immunity (25). Probiotics are recommended as valuable alternative to antibiotic therapy in order to avert potential harm to commensal bacteria and antimicrobial resistance (2, 64). Probiotic strains including *Lactobacillus delbrueckii* subsp. *lactis* DM8909, *L. gasseri* Lba EB01-DSM 14869, *Lactobacillus rhamnosus* GR-1, *Lactobacillus fermentum* RC-14, *Lactobacillus reuteri* RC-14, *Lactobacillus casei* subsp. *rhamnosus* (Lcr35), *Lactobacillus brevis* CD2, *Lactobacillus salivarius* FV2, *Lactobacillus acidophilus* KS400 and *Lactobacillus plantarum* FV9 have been used orally or intravaginally in the treatment of bacterial vaginosis (BV) (65, 66) and gastrointestinal disorders as they can improve urogenital and gastrointestinal health through immune modulation, pathogen displacement, and establish an environment less conducive to proliferation of pathogens and virulence factors (66). Lactobacilli and bifidobacteria of intestinal origin show antimicrobial properties through interfering with other bacteria and by producing antimicrobial substances such as bacteriocins. These properties have informed their use in combination as probiotics against *Helicobacter pylori*, *E. coli*, *Salmonella*, *Listeria monocytogenes* and rotavirus infections both in human and animal models. An example is the VSL#3, a multispecies probiotics mixture containing *L. casei*, *L. plantarum, L. acidophilus* and *L. delbrueckii* subsp. *bulgaricus*, *B. longum*, *Bifidobacterium breve*, *Bifidobacterium infantis*, and *S. salivarius* subsp. *thermophilus*, used in the treatment of IBD, irritable bowel syndrome, pouchitis, and obesity (20).

The loss and recovery of endogenous vaginal lactobacilli are central to the acquisition and cure of BV respectively. The restoration of endogenous vaginal lactobacilli after probiotics treatment is usually gradual and steady, conferring strong colonization resistance by competitively, biochemically and immunologically replacing pathogens and re-establishing vaginal homeostasis (66, 67). The long-term lower recurrence rate observed in women treated with probiotics (65, 67) could be attributed to their capacity to steadily re-establish vaginal homeostasis (66). A cure rate of 90 (68) and 88% (69) have been recorded with the use of intravaginal *L. rhamnosus* GR-1 and *L. reuteri* RC-14 only, and a combination of antibiotics (metronidazole) and probiotics (*L. rhamnosus* + *L. reuteri*), respectively.

Bacterial vaginosis and vulvovaginal candidiasis (VVC) are common genital tract disorders of reproductive-age women characterized by a reduction in lactobacilli (8). Due to *Lactobacillus* deficiency, there is an increase in vaginal pH, overgrowth, and colonization by anaerobic species such *G. vaginalis* (BV) and *Candida albicans* as in VVC. These dysbiotic conditions can also be induced by hormonal changes that occur during menstruation and menopause (2, 8, 64).

Interestingly, in mice models, it has been posited that a dysbiotic vaginal microbiota as in BV can stimulate a similar phenotype in the gut. For example, *G. vaginalis* infection stimulates activation of NF-κB, TNF-α expression and myeloperoxidase activity in both vagina and colon, while IL-10 expression is suppressed. *G. vaginalis* infection also increase the population of *Firmicutes* and *Proteobacteria* [major lipopolysaccharide (LPS)-producers] and reduce *Bacteroidetes* in the vagina (2, 64). *G. vaginalis* infection stimulate gut microbiota LPS production resulting in dysbiosis characterized by increased *Proteobacteria*-*Bacteroidetes* and *Firmicutes-Bacteroidetes* ratios (2, 64). *G. vaginalis* infection stimulate systemic inflammation including gut inflammation (e.g., colitis). However, probiotics treatment with anti-inflammatory *L. plantarum* (NK3) and *B. longum* (NK49) significantly decreased *Proteobacteria* and increased *Bacteroidetes* populations and consequently inhibited gut microbiota LPS production. This further inhibited LPS-induced activation of NF-κB, TNF-α expression and myeloperoxidase activity, while IL-10 expression was elevated in the vagina and colon. That is, oral administration of NK3 and NK49 reduced *G. vaginalis* infection-induced gut dysbiosis and inflammation. Therefore, by regulating gastrointestinal microbial composition and inflammation, probiotics can mitigate BV *in vivo* (64). Similar experiments in humans are still required.

Another example of gut-vagina microbiota crosstalk is in the case of recurrent BV, which may be due to recolonization of the vagina by BV-associated organisms from the rectum. Women at high risk of recurrent BV may have high densities of BV-associated bacteria in their rectum, which re-infects the vagina after treatment. A similar recto-vaginal translocation may be seen in the susceptibility of some women to group B *Streptococcus* (GBS) and *E. coli* infections (5). The intestinal tract is regarded as the primary source of GBS vaginal infection in pregnant women (70, 71). Additionally, the incidence of BV is lower when *Lactobacillus* spp. dominate both the vagina and rectum compared to when it dominates only one or none of these niches (72). Oral administration of probiotics reduced recurrent BV; however, direct vaginal application e.g., as pessary, may provide more rapid treatment (5, 25).

These observations provide evidence of the existence of crosstalk between the gut and vaginal microenvironments. Oral probiotics can also mitigate BV and pro-inflammatory cytokine expression in the vagina, which can subsequently alter the inflammatory status in the gut by acting on bacteria and immune mediators including macrophages, lymphocytes and dendritic cells. Because of the observation of mixed outcomes with the use of probiotics, it has been postulated that for an effective resolution of severe BV infection, the entire microbiota rather than a single bacterial species is required (10). This has facilitated the use of fecal microbiota transplantation (FMT), which involves direct infusion of fecal suspension (bacteria and metabolites) from a healthy donor into the GIT of an infected recipient in order to restore a eubiotic gut microbiota (2). This is done via colonoscopy, retention enema (transrectally), nasogastric/nasoduodenal tube or esophagogastroduodenoscopy, and has been successful in the treatment of pseudomembranous enterocolitis, *Clostridium difficile* (73) or *Clostridium difficile*-like infections etc. (74–76), with about 91% mean cure rates (76). Because of the relationship between gut and female genital tract microbiota, with the association of specific gut microbiota signatures with female genital conditions including BV, endometriosis and polycystic ovary syndrome (PCOS), FMT may be clinically useful in the management of dysbiosis associated genital tract disorders (2).

Furthermore, as the gut microbiota interventions may be ecologically different from those of a dysbiotic vagina microbiota, the use of vaginal microbiome transplant (VMT) could be more beneficial in treating intractable and recurrent BV. This was recently tested in five BV patients, four of who showed full long-term remission determined by reconstitution of *Lactobacillus*-dominant microbiota and significant improvement of symptoms and Amsel criteria at the end of follow-up at 5–21 months after VMT (10). No adverse effects were recorded in any of the five women. The need for randomized, placebo-controlled clinical trials to ascertain the therapeutic efficacy of this mode of BV treatment has been suggested (10).

### Gut-Vaginal Microbiota-Metabolites Phenotypes

Both the gut and the female genital tract microbiota produce unique fermentation metabolic by-products in conjunction with the host cells. Like the microbiota, these metabolic products can trigger local immunological responses with systemic implications that can influence host susceptibility to several metabolic and inflammatory diseases such as obesity, diabetes (20); IBD (Ulcerative colitis and Crohn’s disease), irritable bowel syndrome, allergies (asthma), and colorectal cancer (56); as well as genital tract disorders such as BV, VVC, aerobic vaginitis, and adverse pregnancy outcomes (8, 70).

### Gut Microbiota-Metabolites

Gut microbiota dominated by *Bacteroides*, *Bifidobacterium*, *Lactobacillus*, and *Akkermansia*; and deficient in *Firmicutes* (*Clostridium*), *Prevotella*, and *Methanobrevibacter*, express high amounts of SCFAs including acetate, propionate and butyrate that are microbiota-induced fermentation products (20). SCFAs are produced in the distal small intestine and colon where saccharolytic bacteria (*Bacteroidetes*, *Firmicutes*, and *Actinobacteria*) ferment non-digestible carbohydrates like resistant starch, dietary fiber, inulin and other low-digestible polysaccharides; and some proteins and peptides through the glycolytic (Embden–Meyerhof–Parnas) and/or the parallel Pentose-phosphate pathways (Figure 2) (77, 78). The proximal colon (cecum) is the primary site of fermentation due to highest availability of substrates and free water (23, 78). Acetate, propionate and butyrate which constitute 90–95% of colonic SCFAs are produced in a molar ratio of 60:20:20 (3:1:1), respectively (23, 79, 80), attaining a combined concentration of 50–200 mM in the human colon (81, 82).

Production of SCFAs lowers the pH of the colon, which influences gut microbiota competition for growth and survival. The health-promoting lactic-acid bacteria (lactobacilli and bifidobacteria) thrive in this low pH at the expense of organisms that cannot tolerate low pH conditions, such as yeasts and opportunistic pathogens *Clostridium* and *E. coli* (77). SCFAs are absorbed by the intestinal epithelium through passive diffusion or carrier-mediated uptake according to their charges. The carrier mechanisms (transporters) include monocarboxylate transporter 1 (MCT1) and MCT4; and sodium coupled monocarboxylate transporter 1 (SMCT1) and SMCT2 (79, 80).

The metabolic and immunity-related effects of SCFAs are mediated by interaction with G-protein coupled receptors (GPRs) or free fatty acid receptors (FFARs), i.e., GPR41 (FFAR3), GPR43 (FFAR2), and GPR109A (HCAR2); and through the inhibition of histone deacetylase (by butyrate and propionate mainly).(77–80) Unlike GPR41 and GPR43 that are located on chromosome 19q13.1 (78, 83) and show affinity for all SCFAs, GPR109A binds to butyrate, D-beta-hydroxybutyrate and nicotinic acid (79–81), and is located on chromosome 12q24.31 (84). The GPRs are expressed on the intestinal epithelium, adipocytes, and immune cells including neutrophils, monocytes, macrophages (splenic, colonic, and bone marrow-derived), B/T-lymphocytes, peripheral blood mononuclear cells (PBMCs), and monocyte-derived dendritic cells (79, 80, 83, 85).

**FIGURE 3:**
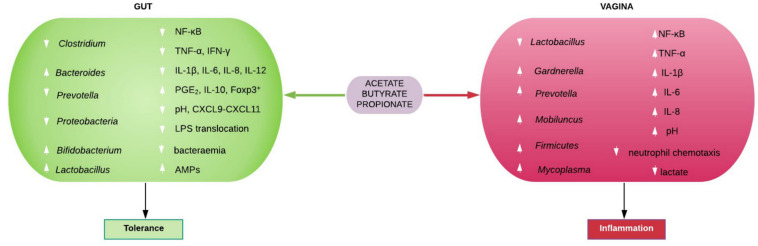
Immunomodulatory roles of gut and vaginal microbiota-generated short chain fatty acids (SCFAs). SCFAs promote eubiosis (including increase in *Lactobacillus* spp. and low pH), tolerance and homeostasis in the gut by suppressing bacterial and lipopolysaccharide (LPS) translocation in to systemic circulation, inhibiting pro-inflammatory chemocytokine production and increase PGE_2_, IL-10, and Foxp3^+^ T cells. In contrast, SCFAs mostly promote dysbiosis (including decrease in *Lactobacillus* spp. and high pH) and inflammation in the vagina. *AMPs*, antimicrobial peptides; *CXCL*, Chemokine (C-X-C motif) ligand; *Foxp3*^+^, forkhead box P3 positive; *IFN-*γ, interferon gamma; *IL*, interleukin; *NF-*κ*B*, nuclear factor kappa-light-chain-enhancer of activated B cells; *PGE*_2_, prostaglandin E_2_; *TNF-*α, tumor necrosis factor alpha.

The physiological functions of the individual gut-derived SCFAs are detailed in the review by Rivière et al. (56) SCFAs maintain intestinal homeostasis by promoting mucus production, stimulating antimicrobial peptides (e.g., β-defensins and REG3γ) production by epithelial cells, increasing intestinal tight junction protein expression, and maintaining intestinal epithelial barrier integrity. Intact gut epithelial barrier prevent bacterial and LPS translocation into the systemic circulation (20, 79, 80, 86). SCFA inhibit systemic accumulation of bacteria (metabolic bacteremia) and LPS (metabolic endotoxemia) that are characteristic of obesity and other metabolic syndrome (56) and chronic inflammatory phenotypes (20). SCFAs also decrease LPS-induced NF-κB activation thereby suppressing NF-κB-mediated expression of pro-inflammatory chemocytokines e.g., TNF-α, IL-1β, IL-6, IL-8, IL-12p40, IFN-γ, CXCL9-CXCL11, etc., and increase the expression of the anti-inflammatory cytokine, IL-10, and Foxp3^+^ CD4^+^ T cells, which turn down immune response (Figure 3) (80).

Short chain fatty acids also stimulate the production of PGE_2_ singly or in combination with LPS in human monocytes and PBMCs. This action could be via EP4 receptor, which is a GPR that maintains intestinal mucosal integrity and inhibit immune response, thereby maintaining intestinal homeostasis (83). GPR43 may also mediate this protective role as they are expressed on colonocytes (83) and show coupling to pertussis toxin-sensitive G_*i/o*_ subunit (78, 79). SCFAs also inhibit LPS-stimulated TNF-α and IFN-γ production in human PBMCs (containing both monocytes and lymphocytes) in a dose-dependent manner. In human monocytes and PBMCs, SCFAs specifically inhibit constitutive monocyte chemotactic protein-1 (MCP-1) and LPS-induced IL-10 production. These indicate that SCFAs inhibit multiple inflammatory mediators, which supports their supplementation through dietary fiber and/or probiotics in the treatment of IBD (83).

The SCFAs also control food intake by increasing the expression of hunger-suppressing hormones such as glucagon-like peptide-1 (GLP-1), peptide YY (PYY), and leptin, which act on the hypothalamus to increase satiety and reduce excess food intake. Therefore, a decrease in SCFAs in the gut is associated with a low-grade chronic inflammation that culminate in obesity and its comorbidities, while an increase indicates eubiosis with health-promoting anti-inflammatory and anti-obesogenic benefits (20).

Further, the SCFAs serve as energy substrates for host cells (77) contributing about 10% of daily energy requirement and are accountable for approximately 75% of energy metabolism in the colonic epithelium (23, 80, 87). Butyrate is the preferred daily source (80–95%) of energy for colonocytes where it is β-oxidized into acetyl-CoA, and large quantity of ATP through the tricarboxylic acid cycle (23, 77, 78, 80). In conditions where significantly higher acetate/butyrate ratios exist, acetate may equally provide energy to the colonocytes (77). Hence, the rate of metabolism of SCFAs can determine the direction of absorption of digestion end products and overall host energy homeostasis (23).

### *Firmicutes*-*Bacteroidetes* Ratio

The gut microbiota consist of *Firmicutes* and *Bacteroidetes* majorly, followed by *Proteobacteria* and *Actinobacteria*, with *Verrucomicrobia* and *Fusobacteria* contributing negligibly. The *Bacteroidetes* phylum mainly produces acetate and propionate, while the *Firmicutes* phylum are the major producers of butyrate (23, 79). The most abundant bacterium in the intestinal microbiota of healthy adults is *Faecalibacterium prausnitzii* (Firmicutes phylum, Clostridium class, Ruminococcaceae family) representing 5–20% of the total bacterial population along with *Roseburia* spp. (23, 77), and other Clostridial cluster XIVa species (56). The abundance of *F. prausnitzii* and other butyrate-producing species of the Lachnospiraceae family are markedly reduced in obese (23) and IBD gut microbiota, and in the setting of colorectal cancer (56).

Generally, an increase in the Firmicutes:Bacteroidetes ratio, which results in a decrease in the total amount of the SCFAs is permissive to LPS-induced inflammatory release of chemocytokines, metabolic endotoxemia and increased risk of metabolic disorders including obesity and type 2 diabetes mellitus (T2DM) (20, 23). This is mediated by toll like receptor (TLR) activation and upregulation of intracellular inflammatory pathways like NF-κB and c-Jun N-terminal kinase (JNK, a TNF-regulated kinase). Despite the reduction in *Bacteroidetes*, obese gut microbiota are rich in *Prevotellaceae* (a subgroup of *Bacteroidetes*), and a good source of LPS (23).

In addition to inhibition of pro-inflammatory cytokine release, SCFAs in the gut also modulate immune responses via GPRs by influencing immune cell migration, phagocytosis and induction of apoptosis in several cells including neutrophils and PBMCs (79, 85, 88). Butyrate appear to exhibit more of these anti-inflammatory functions including inhibition of histone deacetylase (78, 80) and induction of Treg cells (81), and has found great use in the treatment of IBD (80, 85, 88), and as an anti-tumor agent (78).

Nevertheless, there are evidence of contrasting inflammatory effects of the SCFAs. For example, butyrate inhibited production IFN-γ by colorectal carcinoma cell lines (89) and activated lymphocytes in rats, while acetate and propionate stimulated an increase (90). All three SCFAs can also promote neutrophil chemotaxis in a dose-dependent manner through the GPR43 (FFAR2) receptor (79, 83, 91). SCFAs interact with GPRs and inhibit histone deacetylase (butyrate > propionate > > acetate) to modulate intestinal epithelial and immune cell functions, favoring an anti-inflammatory state (79). More comprehensive reviews of the role of gut microbiota-derived SCFAs in metabolic, digestive, cardiovascular and overall wellbeing of the host can be found in Chambers et al. (81), Amabebe et al. (20), Tungland (77), and Parada Venegas et al. (80).

### Vaginal Microbiota-Metabolites

The physiological roles of vaginal microbiota-induced SCFAs are not as extensively studied as the gut’s. In contrast to the gut metabolism and immune response, an increase in acetate, butyrate, and propionate in the cervicovaginal space is associated with decreased lactobacilli, decreased lactic acid concentration, increased vaginal pH and high relative abundance of mixed anaerobes. These include *Gardnerella*, *Bacteroides*, *Prevotella*, *Atopobium*, *Mobiluncus*, *Mycoplasma, Ureaplasma, Sneathia, Eggerthella, Dialister, Streptococcus, Leptotrichia, Megasphaera, Finegoldia, Veillonella, Clostridiales* BVAB 1, 2, 3, etc., that are urogenital pathogens seen in BV and other female genital tract disorders (Figure 3) (92–96). However, a species of the *Lactobacillus* genus i.e., *L. iners* is also associated with dysbiotic vaginal microbiota and BV (96).

Female genital tract SCFAs are fermentation products of carbohydrates as well as amino acid catabolism especially by BV-associated anaerobes. The SCFAs found in the vagina include acetate, propionate, isobutyrate, n-butyrate, and isovalerate (85, 96, 97). Lactobacilli (*L. crispatus, L. gasseri, L. iners*, and *L. jensenii*), which are the dominant species in healthy condition and the *sine qua non* of vaginal homeostasis, exclusively utilize sugars such as glycogen and glycogen hydrolyzates (25) as carbon and nitrogen sources to produce lactic acid (8, 93). Intact vaginal amino acids such as glutamate and branched chain amino acids (BCAAs – leucine, isoleucine, and valine) correlate with increased *Lactobacillus* spp. dominance as observed in healthy women (94). Lactic acid producing bacteria also produce BCAAs, high vaginal levels of which is regarded as another hallmark of *Lactobacillus* dominance in healthy state (94). In contrast, altered gut microbiota and associated increased absorption and circulating levels of BCAA is associated with insulin resistance and a fivefold increased risk of T2DM (98). Further, branched-chain fatty acids such as isobutyrate and isovalerate associated with vaginal dysbiosis and BV (85, 96, 97), exclusively originate from BCAAs (78). This could be another evidence supporting the fermentation of amino acids by BV-associated anaerobes leading to reduced BCAA in BV compared to *Lactobacillus*-dominated healthy conditions. Other amino acids including aspartate, glutamine, glycine, phenylalanine, threonine, tryptophan, and tyrosine are also abundant in *Lactobacillus-*dominated vaginal microbiota but low in BV women (93, 94, 96). SCFAs and succinate can be generated from amino acids that can be metabolized to pyruvate (82). BV is characterized by decrease in these amino acids due to increased decarboxylation by anaerobes into biogenic amines such as cadaverine, trimethylamine, putrescine and tyramine, and fermentation to SCFAs, succinate and formate (93, 94, 96).

It is also plausible that BV-associated bacteria can metabolize sugars and lactate produced by lactobacilli to SCFAs that increase vaginal pH and favor their survival and dominance. By anaerobic metabolism *Prevotella* and *Mobiluncus* produce significantly higher acetate, propionate, butyrate, isobutyrate and succinate as metabolic by-products than *Gardnerella* and *Lactobacillus* (99). Through the mutualistic metabolic exchange of ammonia produced by *P. bivia* and amino acids produced by *G. vaginalis*, both bacteria are able to support the growth and survival of each other. Such synergy promoted the production of succinate and isovalerate by *P. bivia*, while *G. vaginalis* produced acetate and lactate. This is characteristic of the polymicrobial synergistic mixture seen in BV (100). Similar to previous studies (92), our unpublished data indicate that *G. vaginalis*, a dysbiosis-associated anaerobe, metabolize ^13^C-labeled glucose and D/L-lactate to acetate, succinate and formate *in vitro*. Similarly, BV-associated *Megasphaera elsdenii* metabolizes lactate to acetate, butyrate and propionate (101, 102), induce maturation of dendritic cells and increased TLR-4-dependent production of IL-1β, IL-6, IL-8, IL-12p40, and TNF-α (101).

Unlike lactic acid and gut-microbiota derived SCFAs, the SCFAs in the vagina, exhibit significantly lesser antimicrobial activities that contribute to a pro-inflammatory vaginal environment. *In vitro* experiments show that at 20 mM, SCFAs induce IL-1β, IL-6, and IL-8 production by PBMCs. Higher levels are toxic to cells, while lower levels (0.02–2 mM) did not stimulate cytokine release (88). The concentration of acetate in the vagina can be as high as 120 mM (85). SCFAs also significantly enhance TLR2 and TLR7 ligand-induced production of IL-8 and TNF-α in a time- and dose-dependent manner. Their pro-inflammatory cytokine production effect is also partly mediated through generation of reactive oxygen species (88). Acetate and butyrate also stimulate MCT-dependent IL-1β production by PBMCs (103). SCFAs can stimulate oxidative burst in neutrophils. Therefore, alone or in synergy with other microbial products, SCFAs can recruit and activate innate immune cells in the female reproductive tract (85, 88).

*Faecalibacterium prausnitzii* and *Roseburia* (another colonic butyrate-producing Gram-positive anaerobic bacteria and member of the phylum *Firmicutes*) (104), are two major proponents of a healthy gut ecosystem (94), weight loss and reduced glucose intolerance (23, 105). The presence of these organisms in the vaginal ecosystem is associated with proliferation and colonization by opportunistic pathogens and dysbiosis. The relative abundances of *F. prausnitzii* and *Roseburia* were higher in women with common genital disorders such as BV, *Chlamydia trachomatis* and vulvovaginal candidiasis compared to healthy women. It was postulated that colonization of the vaginal milieu by these organisms could be reflective of a general translocation of several microorganisms from the gut to the vagina (94). Besides, the gut has been implicated as the initial source of vaginal *Candida* spp. and BV-associated anaerobes and persists as a reservoir of the yeast and anaerobes (5–7, 106). Ceccarani et al. (94) also observed a significant increase in butyrate, propionate and acetate in BV, *C. trachomatis* and vulvovaginal candidiasis positive women who had higher relative abundance of *F. prausnitzii* and *Roseburia* (in addition to the vaginal anaerobes) compared to healthy women. This was consequently associated with decreased lactate and increased vaginal pH, which are characteristic of dysbiosis.

Specifically, elevated acetate and succinate, in contrast to lactate, is capable of incapacitating the anti-inflammatory chemotaxis of neutrophils, monocytes, and other immunocompetent cells (88, 99, 107), and subsequently creating a dysbiotic vaginal environment permissive to the overgrowth of urogenital pathogens associated with infection, inflammation and preterm birth (PTB, <37 completed weeks of gestation). This anti-chemotactic action helps pathogens to evade phagocytosis and could be the reason no polymorphonuclear leukocytes (pus cells) are found in vaginal secretions of women with BV (99). With prolonged and sustained exposure, SCFAs, especially acetate and to a lesser extent butyrate, dysregulated cervicovaginal epithelial cells’ immune response exhibited as elevated basal and TLR-induced production of pro-inflammatory cytokines including TNF-α and IL-8; but dampened release of IL-6, RANTES, and CXCL10 (108). Consequently, high vaginal acetate concentrations have been employed as a marker of increased risk of spontaneous PTB especially in women presenting with symptoms of labor (109, 110). This predictive capacity is improved when elevated acetate is combined with reduced lactate and glutamate, and increased production of pro-inflammatory mediators such as IL-6 and TNFr-1 (111).

Interestingly, *L. jensenii*, which was associated with higher vaginal pH and PTB (by more than twofold) in our predominantly Caucasian population of pregnant women studied at 20–22 and 26–28 weeks of gestation (112), produced high amounts of acetate and succinate in our recent *in vitro* experiments compared to *L. crispatus* (*under review*). As elevated acetate was able to distinguish those women in this population who had preterm labor (i.e., frequent uterine contractions and <3 cm dilated cervix before 37 weeks) and eventually delivered preterm (109–111, 113), *L. jensenii*’s ability to produce high amounts of acetate may represent another important molecular mechanism in the pathophysiology of infection-associated spontaneous PTB that requires in-depth investigation.

## Conclusion and Future Directions

The vaginal microbiota evolved through a continuous translocation of species from the gut to the vagina or through a mother-to-child transfer during delivery. Though the organisms retain their physio-biochemical characteristics while in the vagina, the immune responses elicited by their metabolic by-products appear to be at variance with those in the gut. This has critical implications for the gynecological, reproductive as well as overall wellbeing of the host and by extension her offspring.

The homeostatic and immunomodulatory effects of SCFAs in the gut are better understood compared to the vagina. The SCFAs (weak acids, pKa 4.8) are present as organic anions in the normal colonic lumen. Their concentration is negatively correlated with the pH of the gut lumen (114). The normal pH of the colonic lumen ranges from 5.5–7.5 in the cecum to 6.1–7.5 in the descending and rectosigmoid colon (114, 115). Butyrate-producing bacteria (*F. prausnitzii* and *Roseburia* spp.) and butyrate concentration decrease as the pH increase from 5.5 in the proximal colon to 6.5 in the descending colon where fermentable dietary fibers are limited. However, there is a corresponding increase in acetate and propionate-producing bacteria (*Bacteroidetes*) (23, 77). Altered SCFA production can stimulate an immune response with loss of epithelial barrier function, bacterial and LPS translocation into systemic circulation, activation of NF-κB and production of high amounts of pro-inflammatory chemocytokines. This is characteristic of the low-grade inflammatory process observed in several metabolic and inflammatory diseases such as obesity, T2DM, IBD, allergies etc. Therefore, elevated SCFAs in the gut appear generally protective (anti-inflammatory) maintaining homeostasis and tolerance between gut epithelium, diet, and commensal microbes (20, 78).

Elevated gut SCFAs during pregnancy may remotely reduce the risk of infection-inflammation associated spontaneous preterm birth. SCFAs maintain intestinal epithelial integrity and prevent leakage of bacteria and LPS into the systemic circulation (leaky gut). This reduces eventual hematogenous spread of bacteria and LPS to the uterus, placenta or amniotic cavity, thereby, preventing the production of LPS-induced inflammatory mediators and prostaglandins (59), that trigger the pathway to parturition. This could be a mechanism underlying the association of overweight and obesity and spontaneous preterm labor and birth, as gut microbiota SCFA production is negatively correlated with body mass index (116). Further, gut microbes have been identified in the amniotic fluid of women that experience preterm premature rupture of membranes. The link between dysbiotic gut microbiota and risk of spontaneous preterm birth requires further investigation as this could explain why treating vaginal infections in some women do not reduce the risk of delivering preterm.

**FIGURE 4:**
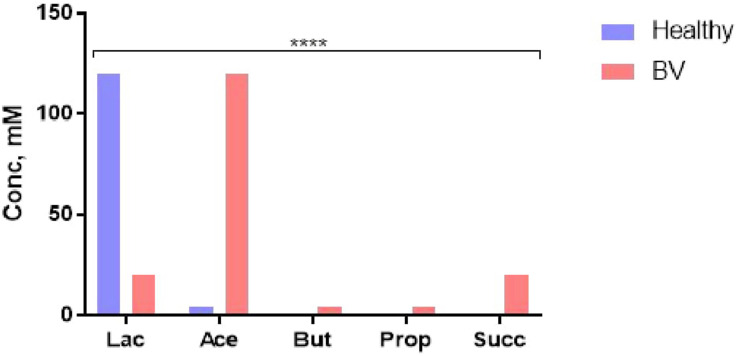
Cervicovaginal fluid metabolite profile in healthy condition (eubiosis) and infection (bacterial vaginosis, BV). *Lac*, lactate; *Ace*, acetate; *But*, butyrate; *Prop*, propionate; *Succ*, succinate. Data Source: Aldunate et al. (92). ****p < 0.0001 (χ^2^ test).

The microbiota-metabolite phenotype and the functional characteristic of the normal vaginal ecosystem is quite different. Lactate (weak acid, pKa 3.9, 10 times more acidic than the SCFAs) produced mainly (∼120 mM) by *Lactobacillus* spp. maintains the vaginal pH at 3.5–4.5. This pH and other antimicrobial activities of lactate and lactobacilli are sufficient to prevent the overgrowth and colonization by opportunistic pathogens including those that may translocate from the gut (8, 25, 92, 94), maintaining the SCFAs at low concentrations (Figure 4) (92). Increase in vaginal SCFAs and concomitant decrease in lactate as seen in BV (Figure 4), is a marker of dysbiosis and infection as they increase the pH above 4.5 (a favorable pH in the colon), thereby, encouraging luxuriant growth of pathogens (mixed anaerobes) (92). Further, the pathogens synergistically exploit the dysbiotic environment to propagate ascending intrauterine infection, inflammation, and adverse reproductive outcomes including failure of conception, miscarriage, and preterm birth (8). The metabolites are modulated by the balance between lactobacilli and BV-associated bacteria (96).

The SCFAs are also less anti-inflammatory than lactate (108), with propensity to promote inflammation through inhibition of neutrophil chemotaxis and increased production of pro-inflammatory chemocytokines. That is, while the SCFAs maintain homeostasis and promote tolerance (eubiosis) in the gut, they encourage dysbiosis and inflammation in the vagina especially when the health-promoting lactobacilli are deficient. The clinical implication of this contrast in the immunomodulatory roles of gut and vaginal microbiota-generated SCFAs is still underrated and requires further investigation, especially in relation to female reproductive health and the perinatal/postnatal health of the offspring. Further studies of the microbiomial and metabolite profile interactions of the vaginal econiche as it relates to risk of gynecological/reproductive disorders are required.

## Author Contributions

EA: conceptualization and literature search. EA and DA: original draft preparation, review, and editing. Both authors approved the final version of the manuscript for submission.

## Conflict of Interest

The authors declare that the research was conducted in the absence of any commercial or financial relationships that could be construed as a potential conflict of interest.
